# Organic Semiconducting Nanoparticles for Biosensor: A Review

**DOI:** 10.3390/bios13040494

**Published:** 2023-04-21

**Authors:** Zheng Wang, Dongyang Han, Hongzhen Wang, Meng Zheng, Yanyi Xu, Haichang Zhang

**Affiliations:** 1 Key Laboratory of Rubber-Plastics of Ministry of Education/Shandong Province (QUST), School of Polymer Science and Engineering, Qingdao University of Science and Technology, 53-Zhengzhou Road, Qingdao 266042, China; 2 Department of Environmental Health, School of Public Health, Fudan University, Shanghai 200032, China; 3 R&D Center of Polymer Materials, Qingdao Haiwan Science and Technology Industry Research Institute Co., Ltd. (HWSTI), Qingdao Haiwan Chemistry Co., Ltd. (QHCC), Qingdao, 266061, China

**Keywords:** biosensors, organic semiconducting nanoparticles, in vivo tracking, in vitro tracking

## Abstract

Highly bio-compatible organic semiconductors are widely used as biosensors, but their long-term stability can be compromised due to photo-degradation and structural instability. To address this issue, scientists have developed organic semiconductor nanoparticles (OSNs) by incorporating organic semiconductors into a stable framework or self-assembled structure. OSNs have shown excellent performance and can be used as high-resolution biosensors in modern medical and biological research. They have been used for a wide range of applications, such as detecting small biological molecules, nucleic acids, and enzyme levels, as well as vascular imaging, tumor localization, and more. In particular, OSNs can simulate fine particulate matters (PM_2.5_, indicating particulate matter with an aerodynamic diameter less than or equal to 2.5 μm) and can be used to study the biodistribution, clearance pathways, and health effects of such particles. However, there are still some problems that need to be solved, such as toxicity, metabolic mechanism, and fluorescence intensity. In this review, based on the structure and design strategies of OSNs, we introduce various types of OSNs-based biosensors with functional groups used as biosensors and discuss their applications in both in vitro and in vivo tracking. Finally, we also discuss the design strategies and potential future trends of OSNs-based biosensors. This review provides a theoretical scaffold for the design of high-performance OSNs-based biosensors and highlights important trends and future directions for their development and application.

## 1. Introduction

In recent years, biosensor technology has experienced significant development due to advances in biomedicine and computational science [1,2,3,4]. Biosensors have found widespread use in medicine and biology, from detecting disease biomarkers [5,6] and tracking drug release [7], to ion imaging [8,9,10] and fluorescence imaging in living animals [11,12,13]. These applications have greatly improved the imaging resolution and can assist physicians in making more accurate disease diagnoses. In order to achieve tracking detection in organisms, scientists have developed biosensors with different fluorescence properties [14,15]. These biosensors have the ability to track and monitor specific targets within the body, enabling real-time and precise detection.

Organic semiconductor materials can be excited by various forms of energy, such as light or electricity, which leads to an excited state from the ground state S0 to the first excited state S1, S2, or Sn. However, the excited state is unstable and will return to the ground state via radiation transition and/or non-radiation transition [16]. Herein, radiation transition is typically divided into fluorescence and phosphorescence [17]. Due to their π-conjugated structure, organic semiconductor materials possess semiconductor properties.

**Figure 1:**
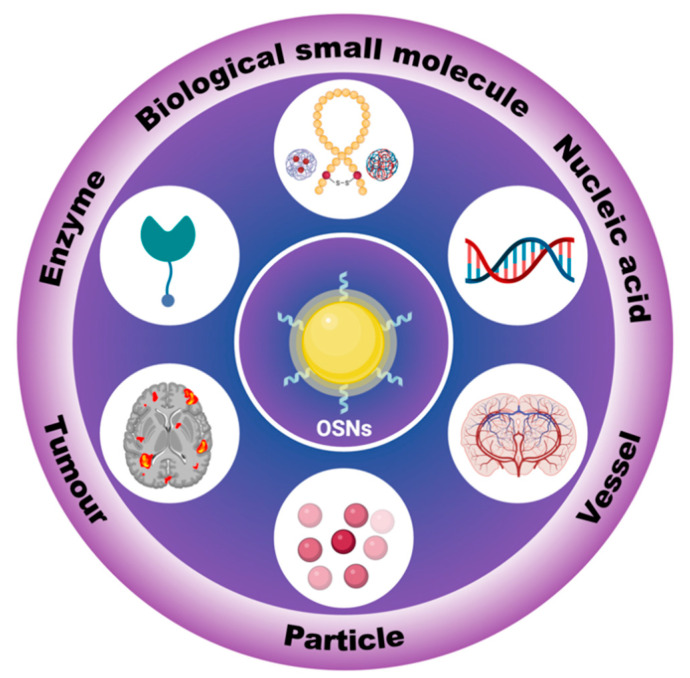
The application of OSNs in biosensors.

Recently, scientists have successfully developed a number of specific recognition fluorescent probes using different strategies, for example, through binding different identifying groups or changing the molecular structure by reaction with the target [18,19]. The organic semiconductor with high biocompatibility is widely used in biosensors [20,21], such as AIE (aggregation-induced emission) based probes. The small size of the organic semiconductor probes makes it easy to penetrate cells [22]. In addition, the outstanding diffusion properties of organic semiconductor probes help improve the resolution of imaging [23]. However, there are still some problems that restrict the development of organic semiconductor probes. On the one hand, organic semiconductor probes usually have poor photostability [24,25]. On the other hand, their toxic effects and metabolism in organisms remain a challenge for organic semiconductor probes application. To solve these problems, researchers have constructed OSNs (organic semiconductor nanoparticles) by attaching or wrapping organic semiconductors in inorganic matrices [26], Metal-organic frameworks (MOFs) [27,28,29], polymers [30], etc. Due to their excellent properties, OSNs are widely used in biosensors (Figure 1).

OSNs are widely used as biosensors for high-resolution tracking in vitro and in vivo [31,32]. Compared to inorganic fluorescent materials, OSNs can be designed with various photosensitive properties and high biocompatibility through different design strategies [33,34]. However, to construct high-performance OSNs for bioimaging, the materials must not only exhibit intense fluorescence, but they must also be designed in a comprehensive manner. For instance, the attachment and wrapping between OSNs and nanoparticles should be stable to prevent detachment [35,36]. Additionally, toxicity is a crucial factor in bioimaging, as some reacting OSNs may be non-toxic before reacting with the target, but they could become toxic when combined with it [37]. Furthermore, the sensitivity and effectiveness of OSN sensors still require further improvements for practical applications.

This review systematically describes the application of OSNs as biosensors in tracking technologies within medicine. Although some articles have reviewed OSNs, only a few have extensively discussed their applications for in vivo and in vitro tracking. Here, we introduce different structures and design strategies based on the tracking targets. Furthermore, we describe different applications of OSNs as biosensors and their potential future applications. The properties and biomedical applications of OSN sensors summarized in this review are listed in Table 1. Ultimately, this review provides a theoretical scaffold for the design of high-performance OSNs and highlights important trends and future directions for their development and application. The properties and biomedical applications of the OSN biosensors summarized in this review are listed in Table 1.

## 2. In Vitro Tracking

By binding of different functional groups or changing the structures of OSNs, we can design specific detecting fluorescent probes. Those probes are widely used for in vitro tracking, which can detect the ions, pH, temperature, amino acid, genetic material, enzyme, and so on in cell models or organizational models. Due to the complexity and potential possible toxic effects of biosensors, they are commonly used for in vitro experiments. It can be well tested for stability and toxicity while tracing in vitro. 

### 2.1. Biological Small Molecules Tracking

Biological small molecules in the body can reveal health conditions, and their detection and tracing are of great importance for biomedical research. Glutathione (GSH), the essential endogenous antioxidant, is the most abundant intracellular nonprotein thiol in mammalian and eukaryotic cells [38,39]. The disturbances in GSH content are generally considered to be associated with various human diseases, such as psoriasis, human immunodeficiency virus (HIV), liver damage, and diabetes [40,41,42]. Given its importance in medicine, achieving highly sensitive detection of GSH, as well as tracking of GSH in living cells, is urgent. Moreover, the tracking of GSH in organisms is favorable for biological investigations and early disease diagnoses [43]. 

**Figure 2:**
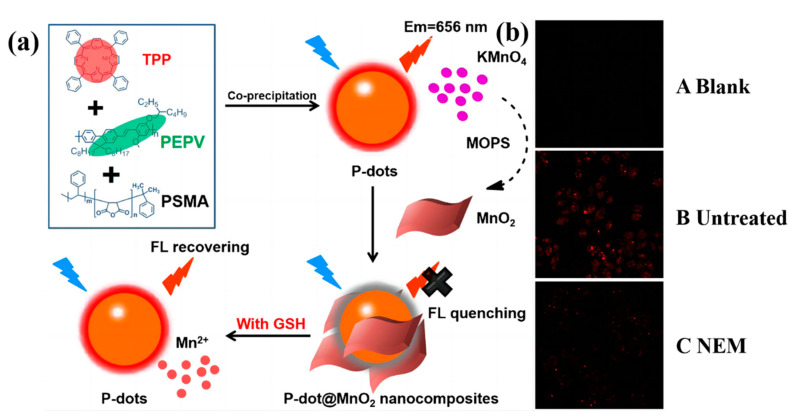
(**a**) Schematic representation of the fabrication of the Pdot@MnO_2_ nanocomposite-based sensing platform for GSH detection. Adapted with permission from [44]. (**b**) Fluorescence microscopic images of (**A Blank**) HeLa cells only, (**B Untreated**) HeLa cells treated with Pdot@MnO_2_ nanocomposites, and (**C NEM**) HeLa cells treated with NEM, as well as P-dot@MnO_2_ nanocomposites. Adapted with permission from [44].

Cheng et al. inserted the tetraphenylporphyrin (TPP) into poly[(9,9′-dioctyl-2,7-divinylene-fluorenylene)-alt-2methoxy-5-(2-ethyl-hexyloxy)-1,4-phenylene] (PEPV) chains and poly (styrene-co-maleic anhydride) (PSMA) to form near-infrared P-dots [44]. Then, they enclosed the P-dots by MnO_2_. The fluorescence of P-dots quench because of the enclosed MnO_2_. However, the MnO_2_ will decompose when meeting the GSH. Thus, the fluorescence can be recovered (Figure 2a). This fluorescence-quenching-fluorescence process makes the highly sensitive detection of GSH with a detection limit of 0.26 μM possible. The author further assesses the potential of P-dot@MnO_2_ as fluorescence imaging probes for monitoring cellular GSH. HeLa cells were incubated with P-dot@MnO_2_, and then the fluorescence images were recorded by a confocal laser scanning microscopy. Figure 2(b A Blank) shows no obvious fluorescence in blank HeLa cells. When the cells were imaged under 458 nm, obvious red fluorescence can be clearly observed. (Figure 2(b B Untreated)) This phenomenon suggests that the membrane-permeable of P-dot@MnO_2_ is remarkable. In order to verify the concentration assessment of GSH, HeLa cells were incubated with the N-methyl maleimide (NEM) (a GSH scavenger 500 μM) to reduce the concentration of GSH. Apparently, the fluorescence intensity was obviously declined with the reduction of GSH. (Figure 2(b C NEM)). This work ingeniously takes advantage of the reaction between MnO_2_ and GSH. However, the mechanism of selective decomposition may cause the run-off of biological small molecules, which will injure the cells or organs in long-term in vitro tracking.

**Figure 3:**
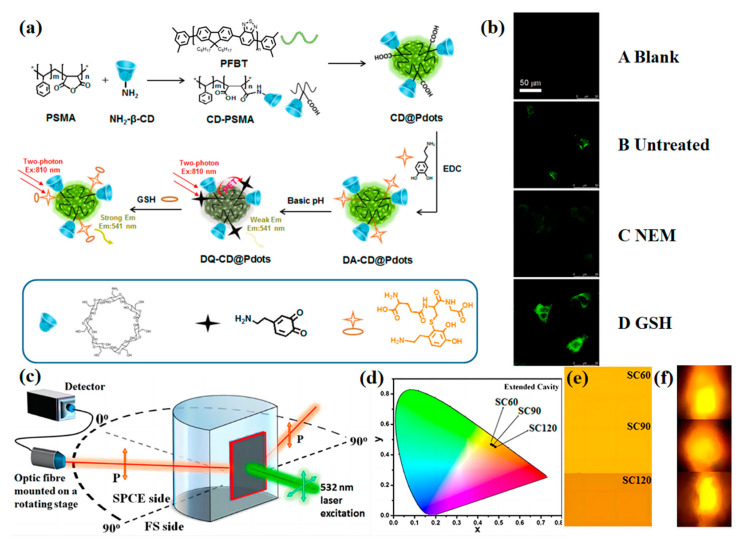
(**a**) Schematic Illustration of the Designed Two-Photon Hybrid Pdots for GSH Sensing. Adapted with permission from [45]. (**b**) Fluorescence microscopic images of (**A Blank**) HeLa cells only, (**B Untreated**) HeLa cells treated with DQ-CD@Pdots, (**C NEM**) HeLa cells treated with NEM as well as DQ-CD@Pdots nanocomposites, and (**D GSH**) HeLa cells treated with GSH as well as DQ-CD@Pdots nanocomposites. Adapted with permission from [45]. (**c**) Optical setup of the SPCE Framework. Adapted with permission from [46]. Fluorescence emission intensities for Ag-SCs (SC60, SC90, and SC120) architectures represented by (**d**) CIE chromaticity plots, (**e**) shade cards, and (**f**) smartphone camera images. Adapted with permission from [46].

Besides the reaction of coating materials, Sun et al., through the structure-changing design strategy build a GSH probe named DQ-CD@Pdots [45]. The DQ-CD@Pdots is constructed from PSMA, β-cyclodextrin (β-CD), poly[(9,9-dioctylfluorenyl-2,7-diyl)-co-(1,4benzo-{2,1′,3}-thiadiazole)] (PFBT), and dopamine (DA). (Figure 3c) The DA molecules are anchored on the surface of the nanoparticles, then the DA molecules are further oxidized to the quinone-like structures (DQ). The DQ is a good electron accepter structure, which can quench the fluorescence of Pdots by intraparticle photoinduced electron transfer (PET) and the “molecular-wire effect”. When the DQ-CD@Pdots combine with GSH, the DQ molecules will be reduced into catechol molecules instantaneously. Thus, the PET phenomenon is inhibited, and the fluorescence will recover (Figure 3(b A Blank, B Untreated)). Thus, the fluorescent intensity will be enhanced or reduced as the concentration of GSH rise (by adding GSH) (Figure 3(b D GSH)) or decrease (by adding NEM) (Figure 3(b C NEM)). In addition, because of the benefits of the highly directional, solid state, and polarized emission of surface plasmon-coupled emission (SPCE) (Figure 3c), scientists have developed some meaningful plasmonics-based GSH sensors. Seemesh Bhaskar et al. [46] reported the silver Soret colloids (Ag-SCs), by changing the adiabatic cooling period, and they constructed SC60, SC90, and SC120 as GSH sensors. The Ag-SCs sensors can achieve femtomolar and high-sensitivity detection of GSH. Significantly, the detection effect can easily acquire by the camera of a smartphone (Figure 3d–f), which can sharply increase the efficiency of clinical diagnosis. This work provides a future development direction and designing guidance for high-performance OSN GSH sensors.

### 2.2. Enzyme Concentration Measurement

Enzymes are widely present in the body and act as biocatalysts. The International Union of Biochemistry and Molecular Biology (IUBMB) classifies enzymes into seven categories, including oxidoreductases, transferases, hydrolases, lyases, isomerases, ligases, and translocases [47]. They all play decisive roles in the activity of the organism. If the activity of enzymes is weakened due to certain defects caused by various factors, it can lead to abnormal reactions, disorders of substance metabolism, and even development of clinical diseases. As a consequence, tracking the concentration and activity of enzymes is of major importance. 

**Figure 4:**
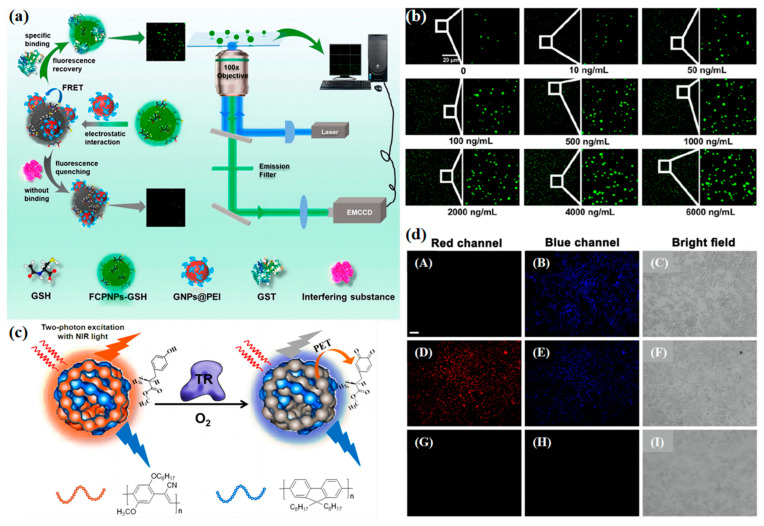
(**a**) Schematic Diagram of the Light Path for Single-Particle Imaging and the Principle of SPE Assay for GST Detection. Adapted with permission from [53] (**b**) Fluorescence images of FCPNPs-GSH on the glass slide surface at various concentrations of GST. Adapted with permission from [53] (**c**) Schematic Illustration of Tyr-OMe Functionalized Pdots for Fluorescence Sensing of Tyrosinase. Adapted with permission from [60] (**d**) (**A**–**C**) Two-photon fluorescence images of B16 cells labeled with Pdots@Tyr-OMe. (**D**–**F**) Two-photon fluorescence images of Pdots@Tyr-OMe-stained B16 cells pretreated with tropolone (50 μM) for 10 min. (**G**–**I**) Two-photon fluorescence images of untreated control B16 cells. RPMI 1640 media supplemented with 10% fetal bovine serum is used for imaging experiments, and the concentration of Pdots@Tyr-OMe is 1.0 μg/mL. The scale bar represents 100 μm. Adapted with permission from [60].

Glutathione S-transferase (GST), which belongs to phase II metabolic enzymes, is crucial for living organisms [48,49,50]. On one hand, GST plays an important role in detoxification. On the other hand, GST can decompose endogenous superoxide radicals by catalyzing the GSH [51,52]. Yameng Han and his co-worker developed a new single-particle enumeration (SPE) method for the sensitive GST assay (Figure 4a) [53]. They synthesize polyethyleniminecapped gold nanoparticles (GNPs@PEI) and glutathione-modified FCPNPs (FCPNPs-GSH). The fluorescence resonance energy transfer (FRET) is formed between the GNPs@PEI and FCPNPs-GSH. Thus, the fluorescence of the system is quenched remarkably. To the contrary, in the presence of GST, the GSH prefers to combine with the GST. Then, the fluorescence emission of FCPNPs-GSH is restored because of the inhibition of FRET. The SPE method is similar to acid–base titration, providing a channel for quantitative measurement of enzyme concentration (Figure 4b) and allowing for sensitive and selective detection of GST with a limit of detection (LOD) of 1.03 ng/mL. 

Tyrosinase (TR) is an important polyphenol oxidase, which can catalyze the aerobic oxidation of tyrosine [54]. The disordering of TR in the human body may be a cause of Parkinson’s disease. In addition, TR has been considered as a mark for melanoma, which corresponds to the concentration and activity of TR [55,56,57,58,59]. Feng Gao’s team used the coprecipitation method to build TR detection pedots with two different fluorescent emissions [60] (Figure 4c). The pedots are combined by poly(9,9-dioctylfluorenyl-2,7-diyl) (PFO) and poly [2-methoxy-5-(2-ethylhexyloxy)-1,4-(1-cyanovinylene-1,4-phenylene)] (CN-PPV), followed by further functionalization with L-tyrosine methyl ester (Tyr-OMe) via electrostatic assembly. As shown in Figure 4d, the TR-catalyzed oxidation product of Tyr-OMe could selectively quench the orange fluorescence emission from CN-PPV Pdots through the PET effect. At the same time, the blue fluorescence emission from PFO Pdots was preserved. This design strategy is very original. However, the electrostatic force is not strong enough, which may induce the reduction of sustainability and leads to detachment in long-period tracking. On this basis, introducing other kinds of intramolecular force, such as hydrogen bonding, can enhance binding between the pedots [61].

### 2.3. Nucleic Acid Concentration Measurement

Nucleic acid concludes deoxyribonucleic acid (DNA) and ribonucleic acid (RNA). It is a biological macromolecular compound polymerized by many nucleotides and is one of the most basic substances of life [62,63,64]. DNA is the main basis for storing, replicating, and transmitting genetic information. Meanwhile, RNA plays an important role in protein synthesis. Thus, facile and reliable methods for the detection of DNA are of vital importance to the medical field, such as medical diagnosis, mutational analysis, gene therapy, biological studies, and specific genomic techniques [65,66].

**Figure 5:**
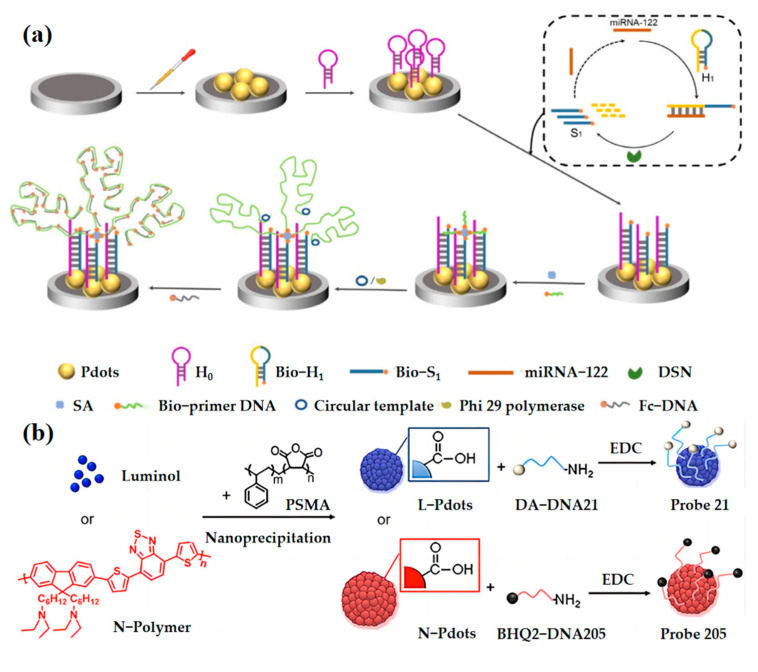

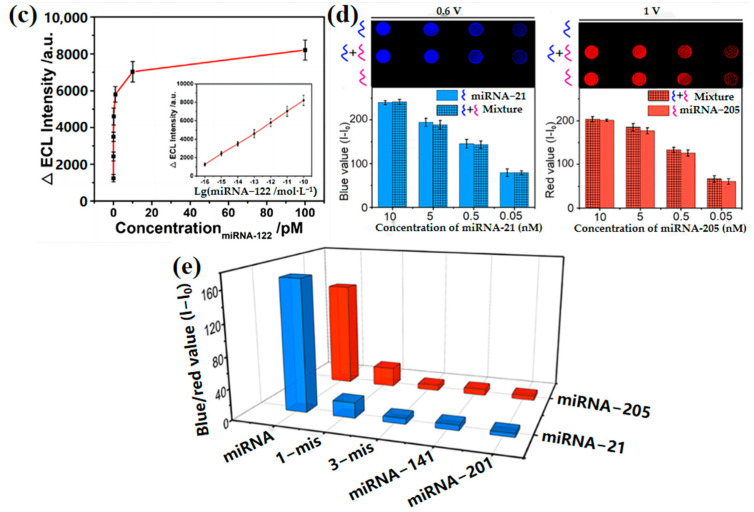
(**a**) Diagram and principle for miRNA-122 detection by the ECL sensor. Adapted with permission from [72]. (**b**) Schematic Diagrams of preparation of Probe 21 and Probe 205. Adapted with permission from [73]. (**c**) The relationship between the ∆ECL intensities and the concentrations of miRNA-122 from 0.1 fM to 100 pM. Inset: the linear relationship. Adapted with permission from [72]. (**d**) Blue and red channels for detection of 10, 5, 0.5, and 0.05 nM miRNA-21, miRNA205, and their mixture of equivalent concentration, respectively. Adapted with permission from [73]. (**e**) Specificity of the proposed imaging method for detection of miRNA-21, miRNA-205, their single-base-mismatched and three-base-mismatched miRNA, miRNA-141, and miRNA-203 at 1 nM. Adapted with permission from [73].

Mi-RNA-122 (microRNA) is a kind of small non-coding RNA, which participates in cell proliferation and apoptosis by regulating gene expression after transcription [67]. An increasing amount of evidence suggests that miRNA-122 is involved in the occurrence and development of various liver diseases [68,69,70,71]. Xu et al. [72] developed an electrochemiluminescence (ECL) sensing platform, which can achieve ultrasensitive detection of mi-RNA-122 (Figure 5a). Firstly, they synthesized a novel polymer with a carboxyl group consisting of fluorene derivate and benzothiadiazole by the nanoprecipitation method. Secondly, they construct the ECL biosensor based on the Pdots for miRNA-122 detection. The system can capture a large number of ferrocene-modified DNA (Fc-DNA). The Fc-DNA can quench the fluorescence of the OSNs by combining them with miRNA-122. In this way, the accurate concentration of miRNA-122 can be determined. This biosensor achieved high-sensitivity detection of miRNA-122 with a wide linear range of 0.1 fM to 100 pM and a LOD of 36 aM (Figure 5c).

Similarly, using the ECL strategy, the Huangxian Ju team [73] developed two miRNA probes named L-Pdot and N-Pdot (Figure 5b) using DA (dopamine) and BHQ2 (black hole quencher 2) as the quencher. Probe 21 can specifically distinguish the miRNA-21, while Probe 205 can distinguish the miRNA-205. The concentration of miRNA-21 and miRNA-25 (Figure 5d,e) can be quantitatively identified through the analysis of (PL) photoluminescence spectroscopy and ECL images. These findings show the potential of the ECL strategy in clinical diagnosis. However, these probes commonly can only detect RNA in cell lysates, and designing in-cell RNA-detecting probes or further in-vivo RNA-detecting probes is very meaningful.

**Figure 6:**
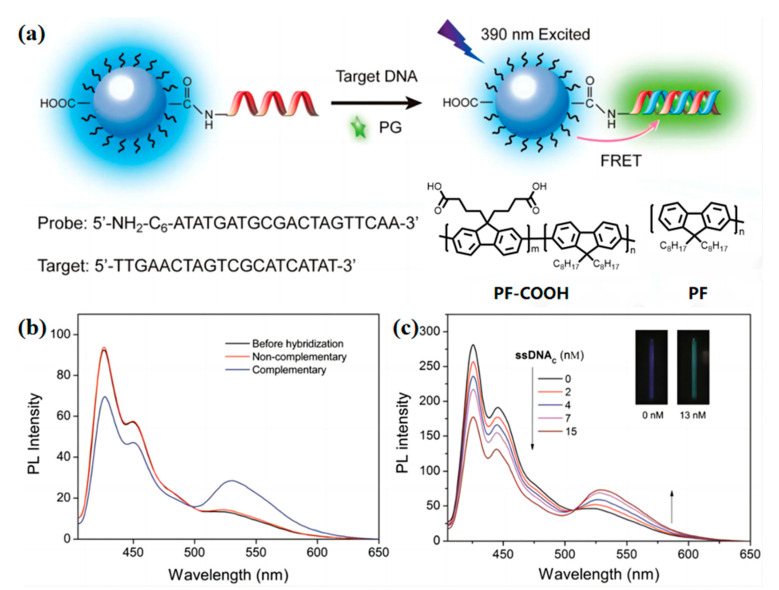
(**a**) Schematic illustration of the PF-DNAP CPNs for label-free DNA detection. Adapted with permission from [80]. (**b**) PL change in PF-DNAP CPNs/PG before and after hybridization between complementary target ssDNAC and non-complementary ssDNANC in HEPES containing 10 vol% serum. [ssDNAC] = [ssDNANC] = 8 × 10^−9^ M. Adapted with permission from [80]. (**c**) PL spectra of PF-DNAP CPNs/PG in the presence of ssDNAC with [ssDNAC] ranging from 0 to 15 nM in HEPES containing 10 vol% serum. Insets: fluorescence photographs of PF-DNAP CPNs/PG with and without ssDNA_C_. λex = 390 nm. Adapted with permission from [80].

DNA carries all of the genetic information for life [74]. In recent years, DNA analysis has attracted many scientists because of its importance in hereditary diseases, clinical diagnosis, and emerging DNA nanotechnology [75,76,77,78,79]. Bao et al. [80] reported a DNA sensor that selectively (label-free) detects target ssDNA in serum. The sensor is formed by the anionic carboxylic acid-functionalized polyfluorenes PFCOOH and the functionalized PF-COOH Pdots (Figure 6a). Then, the dye Picogreen was embedded as the energy acceptor. The fluorescence of Pdots will redshift from 390 nm to 530 nm when the Pdots meet the target DNA. At the same time, the color of fluorescence corresponds from blue to green (Figure 6b). In addition, the PF-DNAP Pdots specifically detected the DNA in serum successfully (Figure 6c). In general, the flexible structure of DNA makes research on DNA probes difficult. This work is meaningful for DNA probes, and more DNA OSNs probes that can specifically detect different kinds of DNA are expected in the future. Moreover, for long-time nucleic acid measurements, the influence on the transcription of DNA/RNA also is a key issue. Tracking nucleic acid helps us further the study in biology and medicine. However, there is a key problem in the designing of nucleic acid probes, especially in in-vivo nucleic acid tracking, and the combination of the probes and nucleic acid may cause abnormal nucleic acid transcription [81]. Thus, the in vivo nucleic acid OSNs probes are possible to solve this challenge, such as by developing corresponding non-destructive probes with regards to removal medicine.

## 3. In Vivo Tracking

Different from the biosensors used for in vitro imaging, the influencing factors for in vivo tracking are more complex. Factors, such as water solubility, diffusion resistance, blood circulation, pH, and temperature, all need to be systematically considered during probe design. Moreover, the probes may be non-toxic to the target but toxic to other cells or organs. Therefore, before applying probe technology for in vivo tracking, in vitro experiments should first be performed to ensure its biosafety. Additionally, the probe should have different metabolism speeds corresponding to different tracking periods. In addition, stability and biocompatibility both are decisive factors for OSNs probes in in vivo tracking [82]. Reasonable molecular designing can improve the stability and biocompatibility of OSNs, such as designing a stable molecular skeleton, reducing halogen atom content, and so on [83,84,85,86]. Moreover, changes in molecular polarity due to changes in molecular structure may change the toxicity of the system.

### 3.1. Tumor Localization

Today, cancer is a major public health problem and the major cause of death globally [87,88]. At present, the main diagnostic methods of cancer include ultrasound imaging (US) [89], single photon emission computed tomography (SPECT) [90,91,92], positron emission tomography (PET) [93,94,95], electronic computed tomography (CT) [96], magnetic resonance imaging (MRI) [97,98,99], and optical imaging [100,101,102]. Among them, radiological imaging has not only unavoidable radiological risks but also certain deficiencies in specificity, sensitivity, resolution, etc. [103,104,105,106]. As a non-invasive technique, fluorescence imaging has the advantages of low risk of harm to humans, high sensitivity, and short response time, thus receiving increasing attention from researchers.

**Figure 7:**
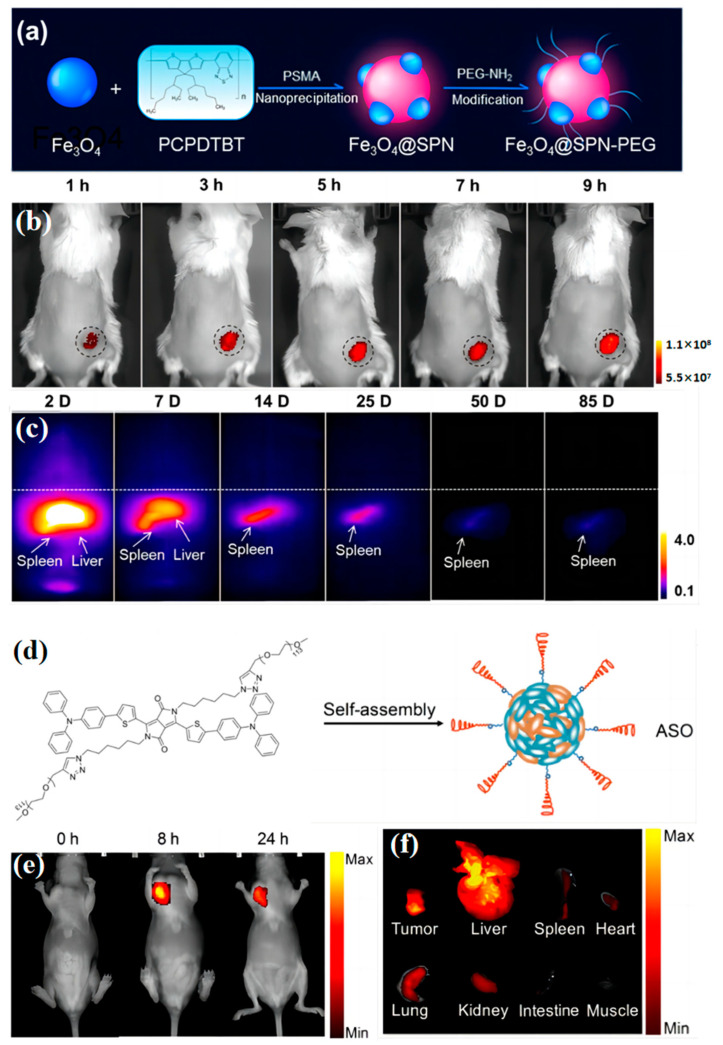
(**a**) Schematic preparation of MMPF NPs. Adapted with permission from [107]. (**b**) Longitudinal MPI images of mice injected with MMPF NPs (2 mg/kg). Adapted with permission from [107]. (**c**) Longitudinal fluorescence images of mice (excitation: 680 nm; emission: 810 nm) after i.v. injection of MMPF NPs (2 mg/kg). Adapted with permission from [107]. (**d**) Illustration of the synthesis of ASO nanoparticles via self-assembly. Adapted with permission from [111]. (**e**) Fluorescence images of a subcutaneous 4T1 tumor in a nude mouse 0, 8, and 24 h after intravenous administration of ASO. Adapted with permission from [111]. (**f**) Fluorescence imaging of major organs of mice 24 h after systemic administration of ASO. Adapted with permission from [111].

Song et al. [107] reported a fluorescent nanoparticle named MMPF NPs (Figure 7a). The MMPF NPs can multimodality locate the tumor in organisms. Combined with MPI, MRI, and photoacoustics to enhance the imaging accuracy of tumors, this MMPF NP has been confirmed as an effective research method. In addition, the half-time of the MMPF NPs in circulation is 49.16 h, and they possess high tumor accumulation (18% ID/g) (Figure 7b). The ultralong half-time and high tumor accumulation mean that the probe has more chances to enter the viscera and accumulate more in the target tumor after injection into the body. Thus, the imaging performance can be substantially improved. Moreover, MMPF NPs offer ultrasensitive MPI imaging of tumors, enabling long-term tracking in mice (nearly three months) (Figure 7c). This work developed a multimodality tracer that has great potential in long-period tumor tracking. However, further medical experiments are needed to determine whether the long-term presence of the probe in the body would cause adverse effects on the health of the organism.

Nanoparticles are commonly prepared by the method of nanoprecipitation. However, the instability of the nanoparticle structure greatly weakens the performance of the biosensor [108,109,110]. Chao Yin and his coworkers reported a new self-assembly method to replace the nanoprecipitation method [111]. They reported non-dissociable near-infrared (NIR)-absorbing OSNs for in vivo PA (photoacoustic imaging) and fluorescence imaging. The OSNs are constituted of amphiphilic semiconducting oligomer (ASO) and hydrophilic poly (ethylene glycol) (PEG) side chains (Figure 7d). The ASO consists of pyrrolidine (DPP) and triphenylamine (TPA). The DPP and TPA both are organic photoelectric molecules with excellent performance, which are widely used in organic field-effect transistors (OFET) [112,113,114], Perovskite solar cells (PSCs) [115], and so on.

In the ASO system, the DPP acts as the accepter (A), while the TPA acts as the donor (D). The D-A design strategy can enhance the carrier transfer rate to improve the strength of fluorescence. Besides, adjusting the lowest non-occupied molecular orbital (LUMO) and the highest occupied molecular orbital (HOMO) levels of the molecules can change the emission wavelength. The ASO and PEG can form water-soluble nanoparticles by self-assembly. At the same time, the PEG side chains can reduce nonspecific interactions with plasma proteins. Thus, the system overcomes the dissociation issue of SPNs. The probe achieves efficient accumulation in the tumor of living mice with high signal-to-background ratio tracking (Figure 7e,f).

### 3.2. Blood Vessel Imaging

In the biomedical field, fluorescence imaging, as a highly sensitive non-invasive imaging technique, poses less risk of harm to the human body and has a shorter response time. It has great potential in organ tracking, especially for deep-tissue diagnosis [116]. X-ray radiography is a common method for gastrointestinal disease diagnosis [117,118,119]. Before the X-ray radiography, the patient needs to take the contrast agent orally, such as in the form of a barium meal. The barium meal will be excreted out of the body by defecation [120,121,122,123,124]. In short-period medical examinations, metabolism is crucial for the contrast medium.

**Figure 8:**
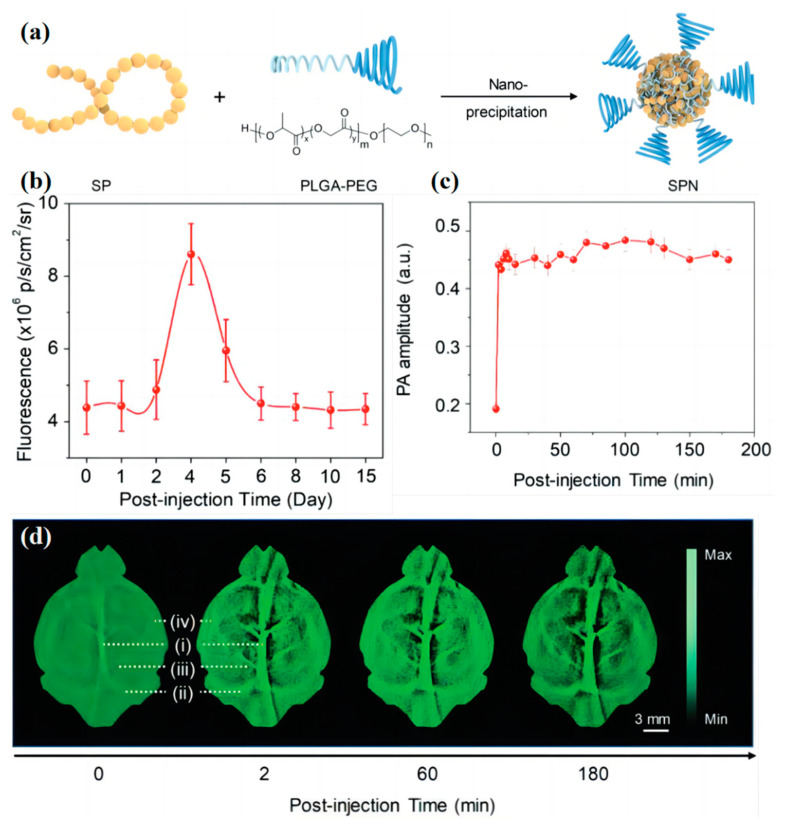
(**a**) Scheme of preparation of NIR-II PA SPNs via nanoprecipitation. Adapted with permission from [125]. (**b**) Quantification of fluorescence intensities of the liver region in living mice as a function of post injection time. Adapted with permission from [125]. (**c**) Quantification of PA amplitudes of major blood vessels in panel. Adapted with permission from [125]. (**d**) PA images of brain vasculature at designated time points at 1064 nm after intravenous administration of SPN-PT (1.1 mg mL^−1^, 1 mL per rat) into living rats: (**i**) superior sagittal sinus; (**ii**) transverse sinus; (**iii**) vascular branches; and (**iv**) middle cerebral artery. Adapted with permission from [125].

Up to now, there were few studies on the biodegradability of biosensors. To solve this problem, Jiang et al. [125] developed the first series of metabolizable NIR-II PA agents named SPN-PT. They combine the hydrolyzable amphiphilic polymer, PA generator, and poly (ethylene glycol)-methyl ether-block-poly (lactide-co-glycolide (PLGAPEG) to form OSNs with great water solubility (Figure 8a), and benzobisthiadiazole (BBT) was selected as the PA generator. BBT is a strong electron-withdrawing monomer, which can extend the system absorbance into the NIR-II region. Because of its excellent water solubility, this material can readily be degraded by phagocytes and be transformed into NIR fluorescent ultrasmall metabolites (≈1 nm). In this way, the OSNs can excrete effectively, leaving no toxicity to organisms (Figure 8b). Furthermore, the SPN-PT overcomes the strong scattering of light by the skull in traditional brain imaging, and brain vasculature can be observed by deep transcranial NIR-II PA imaging (Figure 8c,d).

**Figure 9:**
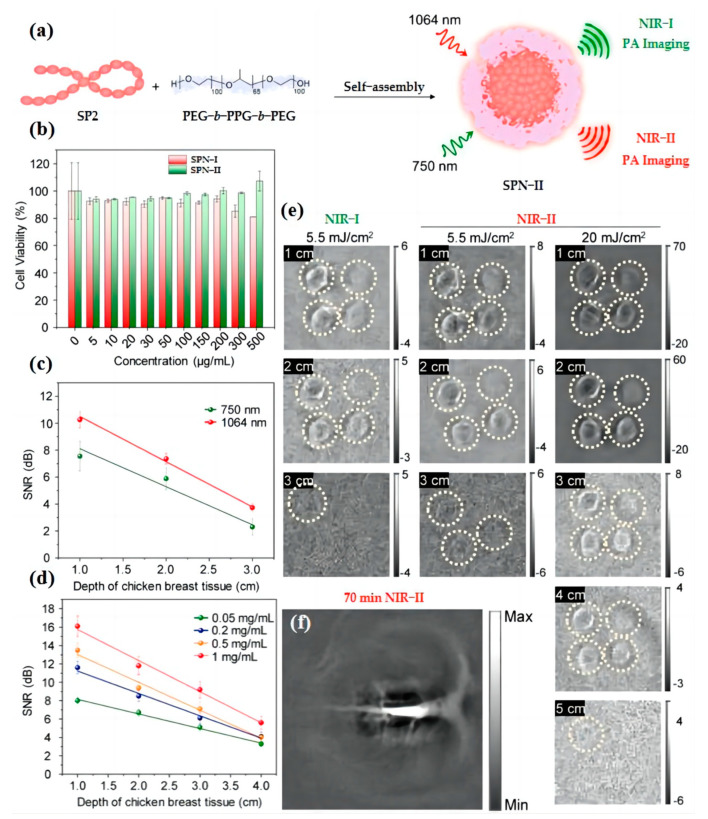
(**a**) Schematic illustration for preparation of SPN-II via nanoprecipitation method. Adapted with permission from [132]. (**b**) Cell viability of NIH/3T3 cells after incubation with SPNs at different concentrations. Adapted with permission from [132]. (**c**) SNR with [SPN-II] = 1 mg/mL at 750 or 1064 nm as a function of the depth of chicken breast tissue. Energy density: 5.5 mJ/cm^2^, R^2^ = 0.92549, and 0.99172 for 750 and 1064 nm, respectively. Adapted with permission from [132]. (**d**) SNR with different SPN concentrations at 1064 nm as a function of the depth of chicken breast tissue. Energy density: 20 mJ/cm^2^. R^2^ = 0.99357, 0.98539, 0.98508, and 0.99005 for 0.05, 0.2, 0.5, and 1 mg/mL, respectively. Adapted with permission from [132]. (**e**) Two-dimensional PA images of the agar gel phantom containing SPN-II solutions were acquired in both NIR windows at different depths. Adapted with permission from [132]. (**f**) PA images of rat cortex at 70 min postinjection of SPN-II at 1064 nm. SPN-II was administered via tail vein injection with a dose of 1.8 mg per rat (*n* = 3). Adapted with permission from [132].

Besides the strong scattering of light by the skull, there are many factors that affect imaging performance, including photon scattering in biological tissues. In recent years, scientists found that fluorescence imaging in the NIR-II window affords reduced photon scattering in biological tissues and lower tissue background [126,127,128,129,130,131]. Thus, further improving the tissue penetration depth is a way to enhance the imaging resolution and imaging fidelity. Jiang et al. [132] reported the first organic imaging agent, named SPN-II (Figure 9a), which can absorb both NIR-I and NIR-II light. In order to compare NIR-II with NIR-I imaging in terms of imaging depth, they add four concentrations of agents into chicken breast tissues with different thicknesses. As shown in Figure 9b–e, the signal-to-noise ratio (SNR) of all depths are obviously higher in NIR-II imaging than that in NIR-I imaging. In addition, the lower energy used for NIR-II allows for imaging of vulnerable areas of the body. Then, the SPN-II is used for in vivo imaging of brain vasculatures in living rats (Figure 9f) Herein, the successful development of the NIR-II imaging system demonstrates its potential for application in high-penetration depth imaging with low damage.

### 3.3. Particles Tracking

**Figure 10:**
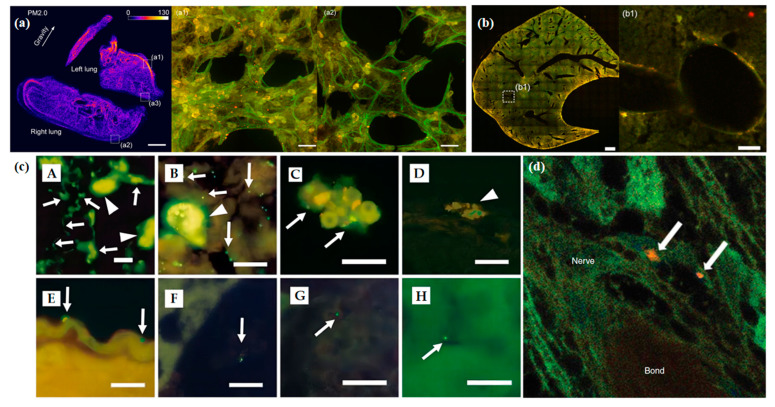
(**a**) Is the deposition patterns of PM_2.0_ and PM_0.2_ in a mouse lung by the fluorescent intensity. (**a1**,**a2**) Show the high and low concentrations of PM_2.0_ particles in two local areas at the left and right edges, respectively, of a mouse lung. Adapted with permission from [135]. (**b**) Is the cross-section of the liver, with the deposition of PM_0.2_ particles. (**b1**) Is the amplified image of the region in (**b**). Fluorescent particles are found on the wall of the lobular sinus of the liver. Adapted with permission from [135]. (**c**) (**A**) The surfaces of the alveolar epithelial cells of mice receiving 20 nm fluorescent particles; (**B**) the alveolar macrophages of mice receiving 200 nm fluorescent particles; (**C**) the red blood cells in the heart of mice receiving 20 nm fluorescent particles; (**D**) the phagocytes on the surface of the endocardium of mice receiving 20 nm fluorescent particles; (**E**,**F**) the red blood cells in the heart of mice receiving 20 nm fluorescent particles; (**G**) the glomeruli of the kidney of mice receiving 20 nm fluorescent particles; (**H**) the glomeruli of the liver of mice receiving 20 nm fluorescent particles. Adapted with permission from [136]. (**d**) Energy excitation-loss fluorescence detection of quantum dots (QDs) in paraffin-embedded nasal tissues. This micrograph illustrates a low level of diffuse false-colour imaging of excitation-loss regions within the nerve fascicles passing through the cribiform plate of the cranium. Two small aggregates of more intense aggregation of QDs (orange) are shown (arrows). TEM images showed the majority of QDs to be within axons. Therefore, QD aggregates are most likely within axons; however, the possibility exists of a periaxonal location as well. Adapted with permission from [137].

Air pollution is an important health concern globally. According to data published by the World Health Organization (WHO), upwards of four million people die early each year due to outdoor air pollution, and the main cause of this is particulate matter pollution [133,134]. Therefore, exploring the deposition of particulate matter in the body and developing imaging techniques for it is a crucial area of cutting-edge scientific research. With the rapid development of nanotechnology, an increasing number of studies are applying biosensors and nanotechnology for tracing particulate matter and in vivo imaging. OSNs, consisting of fluorescently stained polystyrene nanoparticles, are now widely used to model atmospheric particulate matter. In one study, this OSN was successfully used to observe the dynamic deposition of particles in the lungs of mice in real-time using a two-photon microscope [135]. As shown in Figure 10a, benefit from the excellent imaging performance of OSN, the deposition and distribution pattern of particles in the lungs have been tracked. Notably, OSN deposition was observed not only in the lung, but also in the liver, where tissue density is higher and imaging is more difficult (Figure 10b). In addition, using fluorescently stained polystyrene-stained nanoparticles, Furuyama et al. were able to observe good imaging of OSNs in the liver, kidney, spleen, and other organs of mice (Figure 10c) [136]. Quantum dots (QDs) are semiconductor nanocrystals with unique optical and electrical properties. A team of researchers from the United States tracked QDs by using fluorescence and transmission electron microscopy, and this was the first time to observe the migration pathway of QDs from the nose to the brain of mice (Figure 10d) [137]. Previous studies have demonstrated the significant progress achieved in environmental medicine through the use of OSN as a biosensor for in vivo imaging, overcoming the bottleneck that limits high-resolution imaging of particles in vivo. Thus, there is a need to develop different types of OSNs that can simulate natural particles, including PM_2.5_, to explore the impact of particle pollution on human health.

## 4. Conclusions and Outlook

Organic semiconductor nanoparticles (OSNs) offer several advantages for biosensing applications, including strong fluorescence emission, adjustable emission wavelength, and high biocompatibility, among others. Furthermore, by employing different design strategies, we can develop a wide range of functional probes to meet various biosensing requirements. The development of OSNs has the potential to advance the fields of biology and medical science in several ways. Firstly, OSNs can serve as a platform for researchers to monitor the circulation of ions or biological molecules in organisms, thus deepening our understanding of their underlying mechanisms. Secondly, OSNs can be utilized for in vivo tracking to enable faster and more accurate disease diagnosis. Overall, the development of OSNs has the potential to significantly enhance medical science.

Despite several years of development, organic semiconductor nanoparticles (OSNs) are still in the early stages of their development, and further advancements are required. There are several issues that must be addressed to facilitate the continued progress of OSN technology. (i) Although many probes have demonstrated non-toxicity to their targets, their potential toxicity to other organs remains a concern. (ii) More investigations are necessary to identify new applications of OSNs. (iii) The metabolism period of OSNs must correspond to the intended tracking period. (iv) Design OSNs with multiple advantages, including strong fluorescence in the NIR or NIR-II region and good stability. (v) Conducting more clinical trials is essential to validate the accuracy and stability of OSN technology. (vi) The sensitivity and effectivity of OSNs sensors need further enhancement to promote the practical applications of OSNs in clinical diagnoses.

**Table 1:** The important parameters of OSNs biosensors.

Probe	Target	Emission Spectra (λem) (nm)	Excitation Spectra (λex) (nm)	Ref.
P-dot@MnO2	GSH	510	458	[44]
DQ-CD@Pdots	GSH	541	450	[45]
FCPNPs-GSH, GNPs@PEI	GST	500–600	470	[53]
PFO/CN-PPV@Tyr-OMe	TR	586	380	[60]
MiRNA-122 Probe	MiRNA-122	543	-	[72]
L-Pdots	MiRNA-21	425	-	[73]
N-Pdots	MiRNA-205	672	-	[73]
PF-DNAP CPNs	ssDNAC	530	390	[80]
MMPF NPs	tumor	810	680	[107]
ASO	tumor	790	600	[111]
SPN-PT	brain vasculature	820	710	[125]
SPN-II	vessels	1064	-	[132]
